# Leveraging web-based prediction calculators to set patient expectations for elective spine surgery: a qualitative study to inform implementation

**DOI:** 10.1186/s12911-023-02234-z

**Published:** 2023-08-03

**Authors:** Trevor A. Lentz, Byron F. Stephens, Amir M. Abtahi, Jacob Schwarz, Andrew J. Schoenfeld, Bethany A. Rhoten, Shannon Block, Alex O’Brien, Kristin R. Archer

**Affiliations:** 1 Department of Orthopaedic Surgery, Duke University, 300 W. Morgan Street, Durham, NC 27701 USA; 2 Duke Clinical Research Institute, Durham, NC USA; 3 Department of Orthopaedic Surgery, Center for Musculoskeletal Research, Vanderbilt University Medical Center, Nashville, TN USA; 4 Department of Neurological Surgery, Vanderbilt University Medical Center, Nashville, TN USA; 5 Department of Orthopedic Surgery, Brigham and Women’s Hospital, Harvard Medical School, Boston, MA USA; 6 School of Nursing, Vanderbilt University, Nashville, TN USA; 7 Department of Physical Medicine & Rehabilitation, Osher Center for Integrative Health, Vanderbilt University Medical Center, Nashville, TN USA

**Keywords:** Prognosis, Counseling, Decision-making, Expectation, Surgery, Musculoskeletal

## Abstract

**Background:**

Prediction calculators can help set outcomes expectations following orthopaedic surgery, however effective implementation strategies for these tools are unknown. This study evaluated provider and patient perspectives on clinical implementation of web-based prediction calculators developed using national prospective spine surgery registry data from the Quality Outcomes Database.

**Methods:**

We conducted semi-structured interviews in two health systems, Vanderbilt University Medical Center (VUMC) and Duke University Health System (DUHS) of orthopedic and neurosurgery health care providers (VUMC: n = 19; DUHS: n = 6), health care administrators (VUMC: n = 9; DUHS: n = 9), and patients undergoing elective spine surgery (VUMC: n = 16). Qualitative template analysis was used to analyze interview data, with a focus on end-user perspectives regarding clinical implementation of web-based prediction tools.

**Results:**

Health care providers, administrators and patients overwhelmingly supported the use of the calculators to help set realistic expectations for surgical outcomes. Some clinicians had questions about the validity and applicability of the calculators in their patient population. A consensus was that the calculators needed seamless integration into clinical workflows, but there was little agreement on best methods for selecting which patients to complete the calculators, timing, and mode of completion. Many interviewees expressed concerns that calculator results could influence payers, or expose risk of liability. Few patients expressed concerns over additional survey burden if they understood that the information would directly inform their care.

**Conclusions:**

Interviewees had a largely positive opinion of the calculators, believing they could aid in discussions about expectations for pain and functional recovery after spine surgery. No single implementation strategy is likely to be successful, and strategies vary, even within the same healthcare system. Patients should be well-informed of how responses will be used to deliver better care, and concerns over how the calculators could impact payment and liability should be addressed prior to use. Future research is necessary to determine whether use of calculators improves management and outcomes for people seeking a surgical consult for spine pain.

**Supplementary Information:**

The online version contains supplementary material available at 10.1186/s12911-023-02234-z.

## Introduction

Spine-related pain is among the largest drivers of disability and health care costs in the United States [1, 2]. It consistently ranks at or near the top in global reports of disease burden, and data suggest this burden is worsening. In 2016, US health care spending on spine pain was an estimated $134.5 billion (95% CI, $122.4-$146.9 billion), higher than spending attributed to any other health condition that year [1]. Surgery for spine pain is common, growing in incidence [3–5], and accounts for a significant proportion of spine-related health care costs.

Patient satisfaction has become an increasingly important metric by which patients and health care payers like Medicare and Medicaid judge the value of spine surgery [6–8]. Although most patients undergoing spine surgery experience significant benefits, recent work has found that up to 28% of patients are dissatisfied with their surgery despite achieving clinically relevant improvements in pain and function [9–11]. Some of the most important factors driving satisfaction after spine surgery include meeting expectations for return to work and return to previous physical activity [12–15]. Thus, one way to improve satisfaction with surgery is to ensure patients have realistic, evidence-informed post-surgical expectations during the decision-making process [16, 17].

Surgical prediction tools have been developed to guide pre-surgical counseling on patient-specific expectations for surgical outcomes [18]. These tools use patient-level characteristics to determine probable outcomes of surgery across domains such as adverse events (e.g. re-admission)[19, 20] and patient-centered outcomes (e.g. pain, disability) [21–26]. Recently, calculators were developed and internally validated using national data from the Quality Outcomes Database (QOD) [14, 22, 23, 27, 28]. These web-based tools provide individualized risk-adjusted postoperative projections for pain intensity, disability, quality of life, satisfaction, and return to work in patients undergoing elective lumbar and cervical spine surgeries.

Despite their potential value, health care providers have limited guidance on how to best implement prediction tools to facilitate pre-surgical counseling. Therefore, the objective of this study was to conduct interviews with health care providers, administrators, information technology (IT) professionals, and patients to assess barriers, opportunities, and optimal strategies for prediction tool implementation. In particular, we were interested in evaluating topics related to user characteristics, calculator content and interface, workflow integration, organizational culture, and external regulations that would inform implementation strategies for the QOD calculators as well as other prediction tools. We used the Socio-technical Model for Studying Health Information Technology in Complex Adaptive Healthcare Systems [29] as a framework to inform interview content. This 8-dimensional model is specifically designed to address the socio-technical challenges involved in design, development, implementation, use, and evaluation of health IT within complex adaptive healthcare systems. We focused on QOD calculators predicting pain and disability for this project as these outcomes are often of highest importance to patients undergoing spine surgery [9, 30–32], and we expect similar implementation strategies to apply to use of other comparable calculators. We conducted interviews in two health care systems, Vanderbilt University Medical Center (VUMC) and Duke University Health System (DUHS), to compare implementation needs in systems that were familiar and unfamiliar, respectively, with the QOD calculators.

## Methods

### QOD predictive calculators

For this study, we focused on the QOD web-based calculators that predict disability and pain intensity (back/neck and leg/arm) following lumbar and cervical spine surgery. The calculators provide probabilities for (1) any improvement over current level of pain or disability, and (2) an improvement of 30%, which is a valid criterion for minimally clinical important difference (MCID) [22, 23]. Patients enter information about their demographics, symptoms, and pain and disability level into the calculator via a web-based interface. This information is then used to predict each outcome probability. Additional details on calculator content and development are provided elsewhere [22, 23, 33].

### Participant selection

We used purposive sampling to recruit stakeholders located at VUMC and DUHS that met one of the following criteria: (1) surgeon involved in the care of individuals undergoing spine surgery; (2) non-surgeon health care provider involved in the care of individuals undergoing or potentially eligible for spine surgery; (3) health system administrator or health IT faculty or staff involved in the clinical spine care process; (4) patient with low back and/or neck pain consulting with a surgeon. We focused purposive sampling on ensuring a representative sample based on role (for both health care system interviewees) and experience with the calculators (for VUMC health care system interviewees). Purposive sampling of patients focused on gathering a representative sample of interviewees based upon the presence of neck versus back pain. We selected VUMC because familiarity of their health care providers with the calculators allowed us to explore their depth and breadth of experience, with key insights into potential implementation barriers of these specific tools. On the other hand, DUHS interviewees would provide important perspectives on potential barriers and facilitators for clinical sites implementing an entirely novel tool. Institutional Review Board approval was obtained prior to study activities at both sites. All interviewees provided informed consent before participation.

### Data collection and management

Interviews (one-on-one) took place between April and December 2021. A PhD-trained investigator with 2 years of qualitative interview experience (TL, male) conducted all interviews by video call (i.e., Zoom). Provider and administrator/health IT interviews began with a short demographic survey (position/job title, stakeholder group, department, years in practice and at the institution, gender, race, ethnicity, and age). The patient demographic survey included age, race, ethnicity, employment status, level of education, primary diagnosis, acuity of symptoms, history of spine surgery, smoking status and self-reported health status compared to others. The interviewer then described the QOD calculators and showed screenshots of the data entry interface, as well as example outputs from mock data entered into the calculator (Additional File 1). The interviewer used the example outputs to describe how the outputs (i.e., the probabilities of achieving any improvement over current level of pain or disability, and an improvement of 30% over current level of pain or disability) would aid with setting expectations prior to surgery. After this demonstration, the interviewer provided opportunities for the interviewee to ask questions. The interviewer then followed a semi-structured interview guide, informed by the Socio-technical model and customized for each type of interviewee (Additional File 2 and 3). When discussing options for how the calculator should display probability outputs, we tried to minimize bias by showing the same output options to each interviewee, using a standard script, and asking standardized questions as outlined in the semi-structured interview guide. Interviews lasted 30–45 min and were audio recorded, transcribed verbatim, and assessed for data quality before analysis. Key topics covered by semi-structured interviews for each stakeholder type are provided in Table 1.

**Table 1:** Interview Topics^a^

*Topic*	*Description/Example Questions*
**Characteristics of the user**	How do you currently make surgical decisions?
	How can we improve surgical decision-making processes?
	How might the calculator be helpful to patients/providers?
	How would you use the calculator?
**Clinical content**	Do you have concerns for data quality?
	Would this calculator apply to you/your patients?
**Workflow and Communication**	When should the calculator be completed?
	Who should complete the calculator?
	What modifications to the workflow are necessary?
**Human Computer Interface**	How/where should the results be presented?
	What interface should be used (computer, mobile)?
	What additional resources are needed to operationalize the calculator (e.g., scripts, educational material)?
**Internal Organizational Policies, Procedures, and Culture**	What internal policies could impact use?
**External Rules, Regulations, and Pressures**	What external policies (e.g., regulatory, payer) could impact implementation?
	Are there potential harms associated with use of the calculator?
**System Measurement and Monitoring**	How to measures success of implementation or value-added to the system/patient experience?

^a^Based on the Socio-technical Model for Studying Health Information Technology in Complex Adaptive Healthcare System

**Data analysis.** We used a qualitative template analysis approach [34], a form of thematic analysis emphasizing hierarchical coding. Dedoose software Version 9.0.18 was used to organize and analyze interview data. Two team members (TL, BR) read each interview for familiarity. The initial coding template consisted of interview guide prompts and expected categories of responses. TL and BR coded each transcript, and, in an iterative fashion, met to compare codes and reach consensus on coding application after each set of 4–5 interview transcripts were analyzed. Saturation of codes, meaning no new codes or code categories were found in subsequent interviews, occurred after 15 interviews (5 patients; 5 administrators; 5 health care providers) and coders agreed on the analytic template to use for coding subsequently scheduled interviews. We determined that data saturation was achieved with the number of interviews we completed (24 health care provider interviews, 18 administrator/health IT interviews, and 16 patient interviews). We did not plan to conduct a formal comparative analysis of results from VUMC and DUHS interviews, but rather identify where potential implementation challenges converged or diverged between sites that were familiar and unfamiliar with the calculators, respectively.

## Results

Health care provider stakeholders included n = 18 (10 surgeons, 8 non-surgeons) at VUMC and n = 6 (5 surgeons, 1 non-surgeon) at DUHS (Table 2). Administrator/health IT stakeholders included n = 9 at VUMC and n = 9 at DUHS. Patient stakeholders (n = 16) were from VUMC only, with 10 having neck pain and 6 with low back pain. Table 3 provides additional patient demographic and health related information. Tables 4 and 5 provide sample quotes from interviewees. Table 6 outlines actionable strategies to optimize implementation of the QOD calculators in clinical settings based on our holistic synthesis.

**Table 2:** Demographic information for health care provider participants

Health Care System	Variable		Number (proportion) or Mean (range)
**Duke University Health System (n = 6)**	Occupation	Surgeon	5
		Advanced practice provider	1
	Years in practice		25 (6–37)
	Gender	Male	5
		Female	1
	Mean Age (years)		52.5 (45–63)
	Race	White	5
		Other	1
	Ethnicity	Non-Hispanic	6
		Hispanic	0
**Vanderbilt University Medical Center (n = 18)**	Occupation	Surgeon	10
		Advanced practice provider	8
	Years in practice		7 (4–19)
	Gender	Male	12
		Female	6
	Mean Age (years)		41 (35–53)
	Race	White	13
		Other	5
	Ethnicity	Non-Hispanic	16
		Hispanic	2

**Table 3:** Demographic and health related information for patient participants

Variable		Number (proportion) or Mean (range)
Mean Age (years)		62.2 (38–85)
Gender	Male	11 (68.8%)
	Female	5 (31.2%)
Race	White	16 (100.0)
	Other	0 (0)
Ethnicity	Non-Hispanic	15 (93.8%)
	Hispanic	1 (6.2%)
Employment	Employed	7 (43.8%)
	Unemployed	9 (56.2%)
Education	High school diploma or GED	4 (25.0%)
	Two-Year college degree	3 (18.8%)
	Four-Year college degree	5 (31.2%)
	Post-College	4 (25.0%)
Primary Diagnosis^a^	Spinal stenosis	12 (75.0%)
	Other^**a**^	4 (25.0%)
Acuity^**b**^ (months)		72.5 (0.25–600)
Prior spine surgery	Low back	2 (12.5%)
	Neck	1 (6.3%)
	Low back and neck	3 (18.8%)
	None	10 (62.5%)
Location of pain	Neck	10 (62.5%)
	Low back	6 (37.5%)
Self-reported Health Status	Fair	2 (12.5%)
	Good	5 (31.2%)
	Very Good	6 (37.5%)
	Excellent	3 (18.8%)

^a^Options included spondylosis, degenerative spondylolisthesis, isthmic spondylolisthesis, or other; reported by the patient

^b^Number of months patient has reported having low back/leg or neck/arm pain

**Table 4:** Sample health care provider, administrator, and health IT interviewee quotes by topic

Topic		Example Quotes
**Characteristics of the user**	Perceived utility	*I don’t know necessarily that it would change the surgeons chances of offering surgery (Administrator #8)* *It would be a good tool for the provider to say, “This is a tool we use and we don’t think you’re a good surgical candidate and here’s the reason why, and we’ve got numbers to show you. (Non-surgeon health care provider #1)* *People want to hear percentages. I’ve always found that a very difficult question to answer (Non-surgeon health care provider #9)* *There is a large portion that by the time they get to the clinic, they are expecting surgery and they want surgery. And I don’t know that they are going to necessarily care about these numbers. (Non-surgeon health care provider #1)* *I would need to know a lot more about it before I would use it. (Administrator #10)*
**Clinical content**	Quality and applicability of the calculator	*I personally would be totally opposed. But if it were to be that really validated, then I don’t see a barrier. (Administrator #10)* *I would say surgeons should not use a calculation that does not take into account opioid use. (Surgeon #1)*
	Redundancy of information	*Patients checking in are already given [questionnaires], which they’re not really that happy with. So giving them another [set of questions] could be a little overwhelming. (Administrator #8)* *That’s another thing with these patients, if they start seeing repetitive answers, then they just get frustrated. (Non-surgeon health care provider #4)*
**Workflow and Communication**	Selecting who should complete the calculator	*I think it may be more beneficial to do it all across the board from an operations standpoint, because it would be too hard for one person to decipher who needs the calculator and who doesn’t. (Administrator #8)* *It could create a lot of confusion in the patient’s mind if they got a decent predictive number for surgery and we were not offering them surgery. Or the other side, if they were given some sort of horrible predictive value, but they came in and they needed to have surgery. That might make it a bit more confusing. (Non-surgeon health care provider #8)*
	Determining when to administer	*I think that this should be completed in patients, where surgery is being considered an option, but before the decision to pursue surgery has been made. (Surgeon #5)* *If they’re a candidate, but haven’t quite made that decision for surgery or haven’t formally been offered surgery, I think that would be that sweet spot. (Surgeon #12)*
	Getting providers to use it	*It’s going to take some time. They have to get used to the measure. They have to get familiar with it. They also have to see how it behaves in their population. (Administrator #1)* *Generally we’re in the chart a little beforehand, just to look at whatever the past note or imaging before we go into the room. So if [a reminder] is in there, then we’ll go see the patient. (Surgeon #2)* *It’s got to be in their face, at faculty meetings, individual meetings, really making them pay attention to the information so they’ll use it. (Administrator#14)*
**Human Computer Interface**	Communicating probabilities and key terms	*Like the disability score of less than 22, I don’t feel like a patient is going to really know what that means. (Non-surgeon health care provider #3)* *Providing an explanation for how you define disability. Is it your ability to do your activities of daily living? Is it your standing, sitting? Is it those physical movements? (Administrator #14)*
	Resource needs and constraints	*An outcomes coordinator might be a potential resource that would be required to truly make sure that every patient that needs to fill out the survey is filling out the survey and to follow up. (Administrator #9)* *It would definitely require human resources. Any time we ask a patient to either complete it via My Health, someone has to make sure that it’s done. And if it’s not complete, you then have to reach out and get that information. And any time you have human resources dedicated to working, you’re going to have a financial component to that. (Administrator #6)*
**Internal Organizational Policies, Procedures, and Culture**	Culture of research and innovation	*I have found our surgeons to be incredibly open to new in new innovations, especially ones that aren’t going to take a lot of their time. (Administrator #10)* *Because we’re a teaching facility, everybody’s always on board. So that’s a positive and a negative, right? The negative is, because we’re a teaching hospital, there’s always something new, something that we are trying to figure out. So then you have those people who don’t get on board as quickly or as much as you would hope that they would. (Administrator #3)*
	Segmented and siloed care delivery	*The compartmentalization of the different departments and service lines would be an issue. (Surgeon #8)* *The same problem exists here that exists worldwide and creates all of the problems that we all deal with every day, bad communication. (Administrator #14)*
**External Rules, Regulations, and Pressures**	Issues regarding payment	*There may be the opportunity to come into agreements for more expedited approvals of surgeries, if [payers] were aware that we are utilizing this tool. (Administrator #9)* *I would think if this is something that [payers] get their hands on, they’re going to look at it and say, “Well, there’s only a so-and-so percent chance that you’re going to improve. We’re not going to pay for it. (Administrator #4)* *I think a calculator like this could in some instances maybe harm the chances of getting it approved. And then in some cases it might help it. Hard to say (Non-surgeon health care provider #6)*
	Issues regarding liability	*Maybe there should be some sort of disclaimer that this is just a tool that we are using to try to help and should not be something that’s solely relied upon. (non-surgeon health care provider #8)* *I’ve got to be really careful because I’m going to have people second guessing me in the way that I usually don’t use this calculator. (Surgeon #7)*
**System Measurement and Monitoring**	How to measure success	*Does [deciding on surgery] happen quicker when you bring up the calculator? Does that seem to be changing patient behavior on the quicker side to want to move forward with surgery?“ (Administrator #14)* *If you’re not affecting the patient’s decision-making, then you’re useless, because, I don’t think you’re going to affect my decision-making with this. So, you got to find out if it affected their decision, whether or not to have surgery (Surgeon #15)* *The easiest and most superficial way would be to ask a patient, “Did you like that? Did you like having access to that information?“ (Surgeon #6)*

**Table 5:** Sample patient interviewee quotes by topic

Topic		Example Quotes
**Characteristics of the user**	Factors that influence decision to undergo surgery	*Quality of life. Right now there’s just so many things I just can’t do that I normally do. The long-term effects of not having surgery versus surgery, that’s something else I consider. (Patient #11)* *One of the worries for me would be the risks from the surgery itself, infection, paralysis, death. (Patient #16)* *The complexity of what other organs [surgery] might affect, and for me, I define success as being able to have the pain gone, and not to get laid up in the bed (Patient #13)* *You also want to make sure that you’re kind of going through the progression, exhausting some of those less invasive, more conservative options before doing something like surgery. (Patient #14)*
	Perceived utility	*If I had something like this to look at it and somebody told me I had a 20% chance that my back was going to get better, then I would’ve said, “Wait a minute. This is crazy. I’m not doing this.“ (Patient #1)* *In my case, there are unknowns, and if I’m able to define what my chances may be of achieving, some percentage of either disability or better outcomes, that’s a benefit and that would probably weigh in to my decision-making process. (Patient #2)* *There’s probably a small section of people that say, “I don’t want to know. I don’t want to know. I don’t understand numbers.“ And with the graphs and everything, maybe I could see their point. (Patient #5)* *It would save me and [the surgeon] time in making the diagnosis and making the decision, because you’ve already given us the facts pretty plain. (Patient #6)* *I can see it being very helpful for those that are probably a little less interactive with their doctor or their surgeon. (Patient #10)* *It would also talk me out of it if people weren’t getting any, if your reds and greens are reversed, then yes, it would talk me out of it and that’s the information I would like to have, that these people aren’t getting help.(Patient #15)* *Sometimes those discussions [with the surgeon] lead to some uncertainty. I think this would help clear up some uncertainty that you might have after meeting with your surgeon. If you had this information in front of you. (Patient #14)*
**Clinical content**	Quality and applicability of the calculator	*I guess the question is, how this probability is truly calculated. For example, if you’re extremely depressed, does that automatically puts you more towards that only 30% improvement? It would also be helpful to understand, “Oh gosh, if I was less depressed, oh I could whatever, my result could be much better” (Patient #16)* *It’d be helpful if there were other metrics. Like if you did PT for six months, you have this percentage chance of [improvement], or most people only improved 3% or whatever it is. (Patient #9)*
	Redundancy of information or completing extra questions	*I don’t think it [filling out extra questions] such a big deal. The information there’d be very, very nice to have, I would think. (Patient #14)* *Adding extra information after they’ve already done it two or three times [could be a barrier], but when you’re faced with something such as surgery, then you know, it’s a sort of a major deal and I like to have all the information in front of me I could have as far as probabilities and what would take place and what you can expect. (Patient #14)* *I think a lot of this [willingness to complete extra questions] would be determined by how the patient is feeling and what kind of pain they’re in or what kind of discomfort they’re in or what their lifestyle is and what their age is. (Patient #12)*
**Workflow and Communication**	Selecting who should complete the calculator	*Presenting a calculator to me before the surgeon says, “Okay. I think you’re a candidate for surgery,“ is irrelevant. I don’t need this early in the encounter. I would need this when the surgeon says, “Okay. Look, you’re a good candidate for this surgery.“ (Patient #2)*
	Determining when to administer	*I’m a person that likes to plan out and know best case, worst case scenarios. So the more information somebody can give me, even if I’m not at that step right now, like I’m not at a surgery step right now, but I would still like to know the probabilities of positive outcomes for people who do have surgery, 12 months out, five years out, 10 years out. I would love to have that information. (Patient #15)* *I think the initial ask should be a little further out, before the visit, while you’re at home. It gives you time to think through it. You’re not rushed. (Patient #10)*
	Mode of administration	*I would say that for me personally, getting the message or getting the survey through My Health is the best option for me. But of course, you have to have the cell and you have to have email and you have to be willing to open the email. (Patient #5)* *Send it to me in an email. I can look at the questions at home. I can think about them. If you hand me a tablet in a waiting room, and now I’m thinking, “Hey, I’m trying to fill this out.“ Now the doctor will see you now when I’m halfway through or I’m just trying to fill it out quickly. For me, send it to me so I can sit down and I can give you all the correct information and not just go through the motions of checking a block someplace. (Patient #8)* *For me, I’d rather just get a link or do it on my myHealth account, pretty much everything on my computer or tablet or my phone. (Patient #11)* *If it’s me doing it, I get bored if I’m in a waiting room. So that’s like a perfect time to hand somebody an iPad. And if you give me something to do at home with the amount of work emails and cooking dinner, like I’ll never get to it. Like it’ll pop up on my phone and I’m just going to swipe it out of the way. But if I’m sitting somewhere and I’m captive and I have nothing to do, if you give me anything, I’m going to do it. (Patient #15)*
**Human Computer Interface**	Communicating probabilities	*They would need to put a disclaimer on it saying, “This is just average. These are just averages and your results may vary.“ (Patient #5)* *Part of the dialogue should address that, “Here’s what we mean by 30% improvement.“ So, if you have a high level of pain right now, it’ll be fairly significant at a 30% improvement.“ What is improvement, what is the definition of “improvement” versus “30% improvement”? (Patient #10)* *So the thing they need to realize is that these are just averages. Because that’s my thing, if a patient comes in and you tell them, “Well, there’s a 75%,“ they will lock onto that 75% and they are that 75% that’s going to improve. They don’t see the 25% that doesn’t improve. So as a physician or a provider, I would show them this, but I would also like, “These are averages, these aren’t guaranteed. Just always keep that in mind (Patient #15)*
	Description of key terms	*If you have it where you could click on a description [of a key term], then that’s another way. Click here for more information on how to answer this question. Some people are going to take the time to read that and understand it and think about it, and others are just going to blow through and not read those. (Patient #11)* *A lot of those questions are very specific and easy to answer, but some, arm or shoulder numbness, “Well, do I have numbness just because it tingles some of the time? Do I have it at the moment,“ which often is how they’re asking it. That would be a simple answer. You need to be a little more [specific]. (Patient #16)* *Help me understand what moderate disability is or severe or exaggerated symptoms,“ things of that nature. (Patient #2)*
**External Rules, Regulations, and Pressures**	Issues regarding payment	*No, I don’t see how it could be any harm at all. Not to me, I don’t see anything that would be harmful for it at all. (Patient #1)* *I think insurance companies would not do that [use it to deny coverage], nor doctors (Patient #7)* *You may have this mismatch where a patient may say, well, I’m willing to undergo that surgery, even though I was only a 5 or 10% chance because I’m in such pain, whereas a healthcare provider, a surgeon, or even an insurance company may say, well, we’re not going to do this because the probability is so low. (Patient #9)*
	Issues regarding liability	*You’ve got to put your legal hat on and go, “They told me that I had a whatever percent chance.“ You’ve got to put all your disclaimers and all of that kind of stuff. Unfortunately, we live in that kind of a world today where people sue everybody for everything. But I would think the vast majority of people would appreciate something like this. (Patient #11)*
**System Measurement and Monitoring**	How to measure success	*“Were you treated right? Do you feel that the course of treatment is resolving your problem? Is your problem resolved?“ (Patient #5)* *Was the calculator helpful in making your determination to have surgery? I think any surgery patients should have be asked that question in their post survey, to know whether they thought it was a helpful tool. (Patient #9)*

### Characteristics of the user

#### Perceived utility of using an outcomes calculator by providers

Almost all administrators had a favorable impression of the calculators and felt implementation would vary by surgeon depending on whether they found it useful for patient education. Across surgeons, the most commonly stated potential benefits were as a resource to dissuade patients from wanting surgery if they were poor surgical candidates (i.e., no correctable pathology) and to set realistic outcomes expectations. Most surgeons felt the calculators would result in outcome probabilities that were generally similar to what they could surmise through more traditional methods (i.e., talking to the patient, considering imaging results). For this reason, a few surgeons felt the calculators would be of limited use. However, the majority of surgeons reported that having specific, evidence-based probability estimates would help reinforce their clinical impressions while setting appropriate surgical expectations for patients. Non-surgeon health care providers shared these perspectives on the potential benefits. DUHS stakeholders were largely unfamiliar with the QOD calculators and expressed more skepticism about the added benefit of these calculators and concerns over validity of the outcome probability estimates than those at VUMC.

### What is important to patients

We solicited input from patients on what was most important to them when deciding whether to undergo surgery. The most common responses were the probability of returning to work or leisure activities, improvement in pain and function, and symptom severity. Each patient we interviewed had a generally favorable impression of the calculators. Most reported that the greatest value was having quantitative, evidence-informed estimates of outcomes probabilities that would reinforce expectations set by their surgeon. However, some patients stated the calculator would be more helpful if it compared outcomes probabilities for different surgical and non-surgical options.

### Clinical content

#### Quality and applicability of the calculators

The QOD calculators was developed and tested in patients undergoing elective spine surgery for degenerative conditions. A common concern from surgeons was for how well the QOD sample represented their own patient population. These concerns were specific to surgeons that performed complex, or minimally-invasive, spine procedures that were not well-represented in the QOD. Some surgeons felt the calculators did not include all potentially-relevant predictors, like current opioid use. Others felt the utility of the calculators was limited because they do not provide probabilities of improvement with other treatments, and therefore would be unhelpful for setting expectations for surgical versus non-surgical outcomes.

### Redundancy of information

A significant concern across health care providers and administrators was the increased response burden associated with adding questions to the existing battery of questionnaires patients already complete in conjunction with visits. Interestingly, additional response burden was not a major concern for most patients interviewed. While they stated that measures should not be redundant, they were largely in favor of completing calculator items. Patients reported that they would be more likely to complete additional questions if they understood how surgeons would use the information to inform their care.

### Workflow and communication

The biggest concern for providers and administrators was interrupted clinical workflow due to use of the calculators. Interviewees had different perspectives on how to mitigate this concern by strategically selecting who should complete the calculator and when they should complete it.

### Selecting who should complete the calculators

Across health care providers, there was strong consensus that only patients who are already determined to be a surgical candidate should complete the calculators. Providers felt it would otherwise lead to wasted effort, and potentially higher patient demand for unwarranted surgeries. However, many administrators and IT professionals felt that taking a standardized approach to administering the tools to all patients would optimize efficiencies and enhance implementation potential. These stakeholders suggested that patients would only see their results if surgery was deemed suitable.

### Determining when to complete the calculator

We also observed a wide range of opinions on when and how calculators should be administered. Those advocating for universal administration supported completing the calculator at home prior to the initial visit. Links to the calculator would be sent through MyChart or MyHealth interfaces or through email. Clinical staff liked this option because frequent software and hardware challenges (e.g., connectivity issues, depleted batteries) make use of clinic tablets unreliable. Moreover, clinic staff reported that patients would commonly need assistance navigating the tablet interface, further slowing workflow. Most patients also preferred the at-home option because they could complete questionnaires “on their own time, on their own device” and “not when rushed.”

Those advocating for a more targeted approach to administration felt patients should complete the calculator after being identified as a surgical candidate, but before making a decision to undergo surgery. Often, this interval occurs while the patient is in clinic, which introduces the challenge of completing a calculator while minimizing disruption to clinical workflows. One suggestion was to have patients complete the calculator after being triaged as a potential surgical candidate by an advanced practice provider (APP). In most circumstances, the APP and surgeon follow-up visits occur on different days, allowing for an interval in which patients could complete the calculator at home. Another option was to have patients complete the calculators after the visit with their surgeon, but before scheduling surgery. This way, any concerns regarding outcomes could be discussed with the surgeon before scheduling.

A subset of surgeons and non-surgeons expressed strong preferences for completion of the calculator in clinic. They felt patients should only see calculator results while in the presence of a health care provider. The concern was that patients could misinterpret the findings or fail to comprehend how probabilities should inform care decisions. Moreover, some providers had concerns about the quality of data entered into the calculators, noting they would only trust calculator outcomes if they knew patients understood the questions, completed the questions themselves, and were not trying to “game the system” by entering inaccurate responses. Providers felt such concerns were mitigated by having patients complete the calculators in clinic. Regardless of the specific approach, administrators suggested a pilot of the calculators with one or more clinical champions. A pilot would enable health care systems to resolve workflow bottlenecks, identify optimal windows and formats for administration, gather feedback from end-users, and establish IT requirements.

### Human/Computer interface

#### Communicating probabilities and key terms

Interviewees were presented with multiple options for visualizing results. A consensus among all patients and providers was that probabilities should be presented clearly, with explanations of key terms like “disability” and “improvement”. Patients preferred to see their current level of pain or disability and what a 30% improvement would be for each of those outcomes. Another consensus among patients was that results should not be presented using statistical graphs or figures, but in more simplistic forms with whole numbers and concise, layperson descriptions of the outcomes. While patients overwhelmingly supported the potential value of the calculators, some believed the benefit was limited, wanting to know probabilities for higher improvement thresholds like 50% improvement (“30% improvement would not be enough for me”), probability of no pain, or comparison of outcome probabilities across various treatments. Nearly all patients preferred to have a surgeon or other health care provider on hand to discuss results in detail.

### Resource needs and constraints

Most surgeons stated they were comfortable discussing outcomes probabilities with patients. Nevertheless, some stated a script or talking points specific to capabilities and limitations of the calculators could be helpful. Regardless of which patients completed the calculators and when, nearly all providers and administrators agreed that at least some staff resources would be necessary for implementation (e.g., ensure patients have completed the calculator, be on hand to answer questions, remind surgeons to discuss calculator results).

Since the calculators represent new tools for surgeons, we were interested in processes that would help them remember to incorporate results into patient counseling discussions. There was no consensus on the best way to deliver reminders. Some surgeons and their staff preferred pop-up reminders in the electronic health record (EHR), while others were concerned for notification fatigue. A few suggested building reminders into the clinic notes, but qualified that these passive reminders alone would not be sufficient. Few saw benefit in placing flyers around the clinical workspace. The majority of providers felt support staff should be leveraged to remind providers, but that the optimal strategy would vary across clinics and surgeons. One consistent suggestion was that surgeons be reminded of the calculators during regular staff meetings.

### Internal organizational policies, procedures, and culture

#### Culture of research and innovation

Interviewees at both VUMC and DUHS felt the strong research culture and emphasis on quality care in both systems created an environment that would support implementation. Many interviewees stated that practice variability and siloed care delivery across clinics would largely preclude a single institutional implementation strategy. Most administrators and non-surgeon health care providers felt that gaining buy-in from all surgeons would be nearly impossible because some would not see value in the calculators. This observation was supported by skepticism from some surgeons who, as previously mentioned, felt these tools might not apply to their patient population or include the appropriate risk factors. A common opinion across interviewees was that successful implementation would require strong buy-in from Department and Division chiefs.

### External rules, regulations, and pressures

Many providers and administrators expressed concerns that payers could use calculator results to deny coverage. These concerns led some to recommend against documentation of results in the EHR and to suggest that health care systems are clear about which entities will have access to results. Some interviewees raised concerns over whether use of a calculator could expose providers and health care systems to liability if outcomes did not align with calculator results (e.g., continued pain and disability despite high probabilities of success). To address liability concerns, several providers suggested a “warning label” on the calculators and/or confidence intervals for the probability estimates. These additions would ensure patients understood the uncertainty around probability estimates and that results should be used solely as an adjunct to other information when making treatment decisions. Most patients understood the inherent uncertainty in predicting surgical outcomes, but agreed that surgeons should be transparent in discussing calculator strengths and limitations.

### System Measurement and Monitoring

Implementing a new clinical tool consumes time and resources, therefore we were interested in learning how to measure the potential benefits of these efforts. We asked interviewees how they would determine if use of the tools to set outcomes expectations were a “success” in practice. Common metrics noted by health care providers and administrators were improvement in patient satisfaction scores and general improvements in patient management or decision-making from the provider perspective. Health care providers especially valued a reduction in decisional conflict among their patients who may be struggling with the decision to undergo surgery. Patients also reported the potential benefits of reducing decisional conflict, while emphasizing the most important measure of success was the degree to which the calculators accurately predicted their outcomes. Approximately half of the patients stated that improved quality of the patient-provider interaction should be another quality metric by which to evaluate the benefit of the calculators.

## Discussion

This work aims to inform implementation strategies for tools that support patient counseling on expectations and addresses the tension between additional automated tool application and the need for better ways to facilitate shared-decision-making. One strength of this qualitative analysis is the consideration of viewpoints across various stakeholder types at two institutions. We found common potential challenges across institutions, including the need for leadership buy-in, difficulties integrating a web-based tool into existing IT workflows, process variations across clinics that limit integration, and concerns over data quality, liability, and impact on payment. Prior implementation studies of comparable tools in different clinical settings have reported similar barriers [35–38]. While most interviewees agreed the calculators would help to set surgical expectations, they reported widely divergent viewpoints on the best way to implement these tools. These findings suggest that no single implementation strategy would be universally successful, even within the same healthcare system. Nevertheless, actionable strategies emerged throughout our interviews to guide implementation (Table 6). We believe, given the study design, that these determinations have broad capacity for translation in other clinical contexts that are similar to our own.

**Table 6:** Actionable strategies to optimize successful implementation of the QOD calculators

**Actionable strategies targeted toward health care systems** • Clearly articulate the potential utility of the calculators to key stakeholders • Ensure all relevant stakeholders (e.g., surgeons, administrators, clinical support staff, health IT professionals) are involved in implementation decisions for the calculators • Conduct a feasibility pilot to test workflows and identify site-specific implementation barriers • Establish which entities will have access to calculator results • Explore opportunities to integrate the web-based calculators into the EHR interface
**Actionable strategies targeted toward health care providers** • Leverage clinical support staff to assist in reminding surgeons to administer/discuss calculators with patients • Remind surgeons of the calculator during regular staff meetings • Develop simple talking points and scripts for providers to use when discussing the calculators with patients. This should include disclaimers on the limitations of the calculators.
**Actionable strategies targeted toward patients** • Provide patients with clear and concise rationale for why information is being collected and how the calculators may be used. • Develop layperson explanations of key terms, including disability, probability, and improvement. These should be provided along with calculator results. • Limit redundancy of data entry by pulling relevant information from existing data sources (e.g., demographic data from the EHR) when possible • Provide options for patients to complete the calculator at-home and in-clinic

This study explored perspectives across stakeholders in two distinct health care systems that differed in their familiarity with the calculators. Notable similarities across systems include the need for department or division chief buy-in, challenges with integrating a web-based tool into existing EHR and documentation workflows, variation across clinics in workflows and processes that limit integration, and concerns over liability and impact on payment. As with VUMC interviewees, the majority of DUHS interviewees felt that a single implementation strategy was not feasible, but should be tailored to the unique needs, resources, and settings of each clinic and/or surgeon.

Although implementation challenges were similar across systems, the major differences were that DUHS interviewees (1) expressed more skepticism about the validity or accuracy of the calculators and (2) had more doubts about the operational feasibility of assimilating them into care. Most DUHS interviewees stated they would need more familiarity with the calculators before considering their use, and evidence of their benefit in setting expectations beyond current practice. Compared to health care providers, patients more readily identified the value of having access to quantifiable, evidence-based outcomes probabilities. The difference in value judgements between patients and providers on this point highlights an important opportunity to enhance patient-provider communication for surgical outcomes expectations. The majority of patients interviewed liked to see evidence-based estimates that supported their surgeons’ recommendations and felt this information would further reinforce their surgical decisions. Health care providers may not recognize or fully appreciate this patient perspective on the calculators’ value.

Our findings suggest a few critical implementation strategies. These include articulating the accuracy and validity of the calculators to providers, demonstrating their operational feasibility, and convincing providers of the potential value they bring in contributing measurable, evidence-based outcomes probabilities to the clinical encounter. Implementation strategies should also address concerns over who would have access to calculator results and how they could influence payment and liability, as interviewees at both sites raised these issues. Such efforts may include piloting the calculators with one or more clinical champions to provide “proof of concept” regarding benefits, a recommendation raised by a few administrators.

Many providers and administrators expressed doubt that patients would be willing to complete additional questions associated with the calculators. On the contrary, most patients were willing to complete the calculators as long as questions were not redundant and providers made it clear how the information would direct care. This feedback has two main implications. First, it suggests the need to limit redundancy of data entry by using information from existing data sources to populate calculator responses when possible (e.g., importing demographic or diagnostic data elements from the EHR). Second, this feedback reinforces the importance of clearly communicating the intent of clinical data collection to improve response rates and quality of entered data.

Perhaps the most important perceived driver of implementation success is the ease in which patients and providers can use the calculators during routine clinical care. Given the inherent variability in clinical workflows, successful implementation of the calculators at scale would require flexibility on who completes them, and when and how they are completed based on clinic resources, surgeon preferences, and patient needs. Strategies to enhance ease of use for patients includes providing flexible options for them to complete the calculator at-home or in-clinic, and having readily available explanations of key terms (e.g. disability, probability, and improvement). For providers, ease of use would be enhanced by integrating the calculators into the EHR, leveraging clinical support staff to remind surgeons to administer/discuss calculators with patients, receiving reminders about the calculators at staff meetings, and developing simple talking points or scripts to facilitate discussion with patients. The perceived optimal timing of when surgeons discuss probabilities with patients was highly variable and a very small proportion stated they were not interested in outcomes probabilities at all. Thus, health care providers should talk with patients about this resource and their preferences for its use in discussions about surgery.

This work has some limitations. First, we conducted interviews in two academic health systems. The characteristics of these systems and interviewees may not be generalizable to all healthcare settings. Additionally, this study evaluated the implementation potential of specific calculators. While most calculators share similar attributes, we do not know whether the challenges and implementation strategies we identified would apply to other calculators currently available, such as calculators that predict return to work [26].

A second limitation is that our sample of patients had limited diversity in race and ethnicity. We only recruited patient participants from one center. Therefore, the views of these patients may not generalize to more diverse populations in other clinical contexts. Our sample was similar in level of symptom acuity to studies on spine surgery populations using QOD data [14, 22, 23, 27, 28], however tended to be older (median age = 60 years, mean age = 62.2 years) compared to QOD studies (mean ranges 51–55 years). Our sample also had higher levels of education, a higher proportion of male participants, and was under-represented by non-White and Hispanic participants compared to published QOD studies. These comparisons are important because they suggest our study did not fully capture perspectives of younger, non-White, and Hispanic participants undergoing spine surgery, or those with lower educational attainment. These patients may have different communication, informational, or access needs. They may see different value in the calculators, have differing perspectives on the burden of extra questions, want information earlier or later in the care episode, or value information on other outcomes. Studying needs specific to these under-represented populations would further inform calculator implementation for an important care-seeking group. Finally, sample recruitment was driven by a desire to develop a representative cohort of interviewees, not necessarily by the desire for theme saturation. Although we did see similar and consistent responses across interviewees with similar roles, a larger sample may have uncovered additional perspectives.

Our findings have important implications for future work on the topic. Most notably, the calculators studied provide outcomes probabilities as the result of surgery, however are unable to provide outcomes probabilities for other non-surgical treatment options. Multiple patient interviewees noted the importance of comparing outcomes probabilities among different treatment options. To our knowledge, no single resource exists that directly compares personalized outcomes probabilities of different treatments for spine pain. Future work should focus on developing these resources, as well as calculators that predict longer-term outcomes.

Second, future research should focus on understanding how implementation strategies may differ across diverse healthcare settings and populations. This would include determining optimal strategies for communicating key terms (e.g., outcomes probabilities) to patients with differing levels of education and health literacy. It would also entail determining best ways to educate patients and providers on the limitations of the calculators, i.e., that they cannot be used to compare outcomes probabilities for different treatments. Our work also lays a foundation for future effectiveness studies. Specifically, it outlines strategies to ensure successful implementation of these tools and optimize fidelity to protocols that use these tools in pragmatic trials. Such trials are necessary to determine whether use of calculators improves management and outcomes for people seeking a surgical consult for spine pain.

A final direction for future work is to ensure that prediction tools, such as surgical outcomes calculators, do not lead to inequitable care by creating bias in clinical decision-making. This is a major concern as health care systems leverage new technologies and large, population-based datasets to create decision-support algorithms [39–41]. Some surgeons in this study raised concerns that the calculators may not apply to their specific patient population. This concern highlights an important principle; that outcomes probabilities will be most accurate when surgeons use calculators in a patient population similar to the one used to develop the calculator. Any future calculator refinement for more specific populations should pay careful attention to design and take a thoughtful approach to mitigate any potential biases.

## Conclusions

Interviewees had a largely positive opinion of the QOD calculators, believing they would aid in setting appropriate expectations for spine surgery outcomes. Implementation strategies will need to vary according to clinic resources, surgeon preferences, and patient needs. The most successful strategies will provide flexible options for where and how patients complete the calculator, using existing EHR data elements when available to minimize response burden. To encourage calculator use by surgeons, implementation strategies should leverage clinical support staff to assist with administration, include reminders during regular staff meetings, provide scripts or talking points to aid in patient education on outcome probabilities, and address any concerns over data quality, liability, and impact on payment. Future research is necessary to determine whether use of calculators improves management and outcomes for people seeking a surgical consult for spine pain.

### Electronic supplementary material

Below is the link to the electronic supplementary material.

Supplementary Material 1

Supplementary Material 2

Supplementary Material 3

## Data Availability

The datasets generated and/or analyzed during the current study are available from the corresponding author on reasonable request.

## Abbreviations

QOD: quality outcomes database
EHR: electronic health record
APP: advanced practice provider
IT: information technology
VUMC: Vanderbilt University Medical Center
DUHS: Duke University Health System

## References

[1] Dieleman JL, Cao J, Chapin A, Chen C, Li Z, Liu A (2020). US Health Care spending by Payer and Health Condition, 1996–2016. JAMA.

[2] Institute of Medicine (US). Committee on Advancing Pain Research, Care, and Education. Relieving Pain in America: A Blueprint for Transforming Prevention, Care, Education, and Research [Internet]. Washington (DC): National Academies Press (US); 2011 [cited 2020 Apr 28]. Available from: http://www.ncbi.nlm.nih.gov/books/NBK91497/. 22553896

[3] Deyo RA, Mirza SK, Martin BI, Kreuter W, Goodman DC, Jarvik JG, Trends (2010). Major Medical Complications, and charges Associated with surgery for lumbar spinal stenosis in older adults. JAMA.

[4] Ragab A, Deshazo RD (2008). Management of back pain in patients with previous back surgery. Am J Med.

[5] Weiner DK, Kim Y-S, Bonino P, Wang T (2006). Low back pain in older adults: are we utilizing healthcare resources wisely?. Pain Med.

[6] Centers for Medicare & Medicaid Services (CMS) H. Medicare program; hospital inpatient value-based purchasing program. Final rule. 2011. 21548401

[7] Sciubba DM, Pennington Z, Ehresman J. Guest Editorial: Predictive Analytics, calculators and cost modeling in spine surgery. Global spine Journal. Volume 11. SAGE Publications Inc; 2021. pp. 4S–6S. 10.1177/2192568220977185 PMC8076808 33890809

[8] Damberg CL, Sorbero ME, Lovejoy SL, Martsolf GR, Raaen L, Mandel D (2014). Measuring success in Health Care Value-Based Purchasing Programs: findings from an Environmental scan, Literature Review, and Expert Panel Discussions. Rand Health Q.

[9] Sivaganesan A, Khan I, Pennings JS, Roth SG, Nolan ER, Oleisky ER (2020). Why are patients dissatisfied after spine surgery when improvements in disability and pain are clinically meaningful?. Spine J.

[10] Asher AM, Oleisky ER, Pennings JS, Khan I, Sivaganesan A, Devin CJ (2020). Measuring clinically relevant improvement after lumbar spine surgery: is it time for something new?. Spine J.

[11] Khan I, Pennings JS, Devin CJ, Asher AM, Oleisky ER, Bydon M (2021). Clinically meaningful improvement following cervical spine surgery: 30% reduction Versus Absolute Point-change MCID values. Spine (Phila Pa 1976).

[12] Hamilton DF, Lane JV, Gaston P, Patton JT, MacDonald D, Simpson AHRW (2013). What determines patient satisfaction with surgery? A prospective cohort study of 4709 patients following total joint replacement. BMJ Open British Medical Journal Publishing Group.

[13] Mannion AF, Junge A, Elfering A, Dvorak J, Porchet F, Grob D (2009). Great expectations: really the novel predictor of Outcome after spinal surgery?. Spine.

[14] Asher AL, Devin CJ, Kerezoudis P, Nian H, Alvi MA, Khan I (2019). Predictors of patient satisfaction following 1- or 2-level anterior cervical discectomy and fusion: insights from the Quality Outcomes Database. J Neurosurgery: Spine Am Association Neurol Surg.

[15] Cha EDK, Lynch CP, Jadczak CN, Mohan S, Geoghegan CE, Singh K (2022). Meeting patient expectations or achieving a Minimum clinically important difference: predictors of satisfaction among lumbar Fusion Patients. Asian Spine J.

[16] Rampersaud YR, Canizares M, Perruccio AV, Abraham E, Bailey CS, Christie SD (2022). Fulfillment of patient expectations after spine surgery is critical to patient satisfaction: a cohort study of spine surgery patients. Neurosurgery.

[17] Eastwood D, Manson N, Bigney E, Darling M, Richardson E, Paixao R (2019). Improving postoperative patient reported benefits and satisfaction following spinal fusion with a single preoperative education session. Spine J.

[18] Lubelski D, Hersh A, Azad TD, Ehresman J, Pennington Z, Lehner K (2021). Prediction models in degenerative spine surgery: a systematic review. Global Spine J.

[19] Caplan IF, Winter E, Glauser G, Goodrich S, McClintock SD, Hume EL (2020). Composite score for prediction of 30-day orthopedic surgery outcomes. J Orthop Res.

[20] Dimentberg R, Caplan IF, Winter E, Glauser G, Goodrich S, McClintock SD (2021). Prediction of adverse outcomes within 90 days of surgery in a heterogeneous orthopedic surgery Population. J Healthc Qual.

[21] Anis HK, Strnad GJ, Klika AK, Zajichek A, Spindler KP, Barsoum WK, et al. Developing a personalized outcome prediction tool for knee arthroplasty. The Bone & Joint Journal. Volume 102–B. The British Editorial Society of Bone & Joint Surgery; 2020. pp. 1183–93. 10.1302/0301-620X.102B9.BJJ-2019-1642.R1 32862678

[22] Archer KR, Bydon M, Khan I, Nian H, Pennings JS, Harrell FE (2020). Development and validation of cervical prediction models for patient-reported outcomes at 1 year after cervical spine surgery for Radiculopathy and Myelopathy. Spine (Phila Pa 1976).

[23] McGirt MJ, Bydon M, Archer KR, Devin CJ, Chotai S, Parker SL (2017). An analysis from the Quality Outcomes Database, Part 1. Disability, quality of life, and pain outcomes following lumbar spine surgery: predicting likely individual patient outcomes for shared decision-making. J Neurosurg Spine.

[24] Curtin P, Conway A, Martin L, Lin E, Jayakumar P, Swart E (2020). Compilation and analysis of web-based Orthopedic Personalized Predictive Tools: a scoping review. J Pers Med.

[25] Tetreault LA, Côté P, Kopjar B, Arnold P, Fehlings MG, AOSpine North America and International Clinical Trial Research Network (2015). A clinical prediction model to assess surgical outcome in patients with cervical spondylotic myelopathy: internal and external validations using the prospective multicenter AOSpine North American and international datasets of 743 patients. Spine J.

[26] White HJ, Bradley J, Hadgis N, Wittke E, Piland B, Tuttle B (2020). Predicting patient-centered outcomes from spine surgery using Risk Assessment Tools: a systematic review. Curr Rev Musculoskelet Med.

[27] Devin CJ, Bydon M, Alvi MA, Kerezoudis P, Khan I, Sivaganesan A (2018). A predictive model and nomogram for predicting return to work at 3 months after cervical spine surgery: an analysis from the Quality Outcomes Database. Neurosurg Focus.

[28] Asher AL, Devin CJ, Archer KR, Chotai S, Parker SL, Bydon M (2017). An analysis from the Quality Outcomes Database, Part 2. Predictive model for return to work after elective surgery for lumbar degenerative disease. J Neurosurg Spine.

[29] Sittig DF, Singh H (2010). A New Socio-technical model for studying Health Information Technology in Complex Adaptive Healthcare Systems. Qual Saf Health Care.

[30] Turk DC, Dworkin RH, Revicki D, Harding G, Burke LB, Cella D (2008). Identifying important outcome domains for chronic pain clinical trials: an IMMPACT survey of people with pain. Pain.

[31] Ostelo RWJG, de Vet HCW (2005). Clinically important outcomes in low back pain. Best Pract Res Clin Rheumatol.

[32] Whitebird RR, Solberg LI, Norton CK, Ziegenfuss JY, Asche SE, Grossman ES (2020). What outcomes matter to patients after joint or spine surgery?. J Patient Cent Res Rev.

[33] Archer KR, Nian H, Khan I, Pennings JS. Streamlining the QOD Web-based Calculator for Clinical Integration: Development and Validation of a Reduced Prediction Model for Lumbar Spine Surgery. Spine. 2022;In Press. 10.1097/BRS.0000000000004358 35905327

[34] Brooks J, McCluskey S, Turley E, King N (2015). The utility of Template Analysis in qualitative psychology research. Qualitative Res Psychol Routledge.

[35] Porter A, Kingston MR, Evans BA, Hutchings H, Whitman S, Snooks H (2016). It could be a ‘Golden Goose’: a qualitative study of views in primary care on an emergency admission risk prediction tool prior to implementation. BMC Fam Pract.

[36] Evans BA, Dale J, Davies J, Hutchings H, Kingston M, Porter A (2022). Implementing emergency admission risk prediction in general practice: a qualitative study. Br J Gen Pract.

[37] Trivedi MH, Daly EJ, Kern JK, Grannemann BD, Sunderajan P, Claassen CA (2009). Barriers to implementation of a computerized decision support system for depression: an observational report on lessons learned in “real world” clinical settings. BMC Med Inf Decis Mak.

[38] Klarenbeek SE, Schuurbiers-Siebers OCJ, van den Heuvel MM, Prokop M, Tummers M (2020). Barriers and facilitators for implementation of a computerized clinical decision support system in Lung Cancer Multidisciplinary Team Meetings-A qualitative Assessment. Biology (Basel).

[39] Obermeyer Z, Powers B, Vogeli C, Mullainathan S (2019). Dissecting racial bias in an algorithm used to manage the health of populations. Science.

[40] Benjamin R (2019). Assessing risk, automating racism. Science.

[41] Bærøe K, Gundersen T, Henden E, Rommetveit K (2022). Can medical algorithms be fair? Three ethical quandaries and one dilemma. BMJ Health Care Inform.

