# Supplementary material for: Leveraging web-based prediction calculators to set patient expectations for elective spine surgery: a qualitative study to inform implementation
Source: BMC Med Inform Decis Mak. 2023 Aug 3;23:149. doi: 10.1186/s12911-023-02234-z (PMC10399016; doi:10.1186/s12911-023-02234-z)

**Additional File 1. Example QOD calculator input interface and outputs shown to participants during interviews**

**
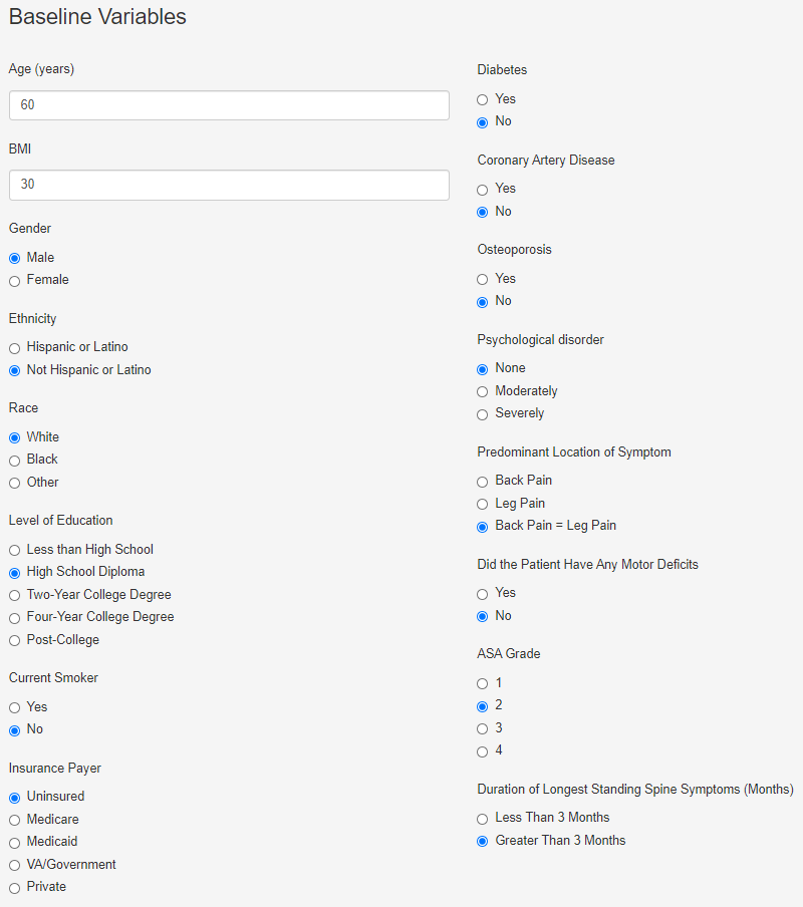
**


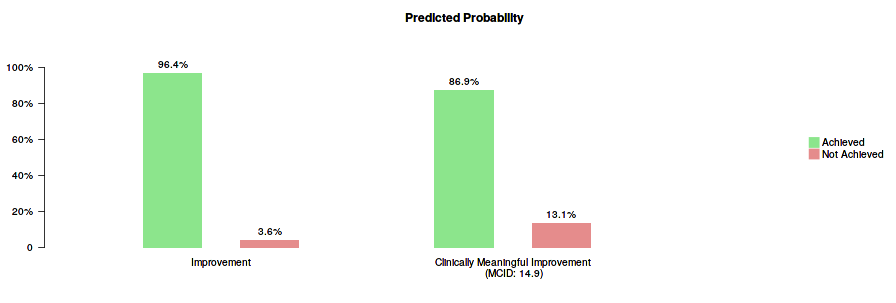

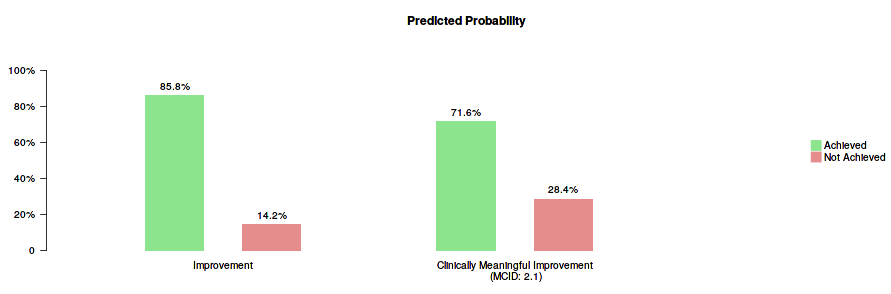


**Probability of improvement in back pain at 12 months**

**Probability of improvement in disability at 12 months**

**Alternative output display options**


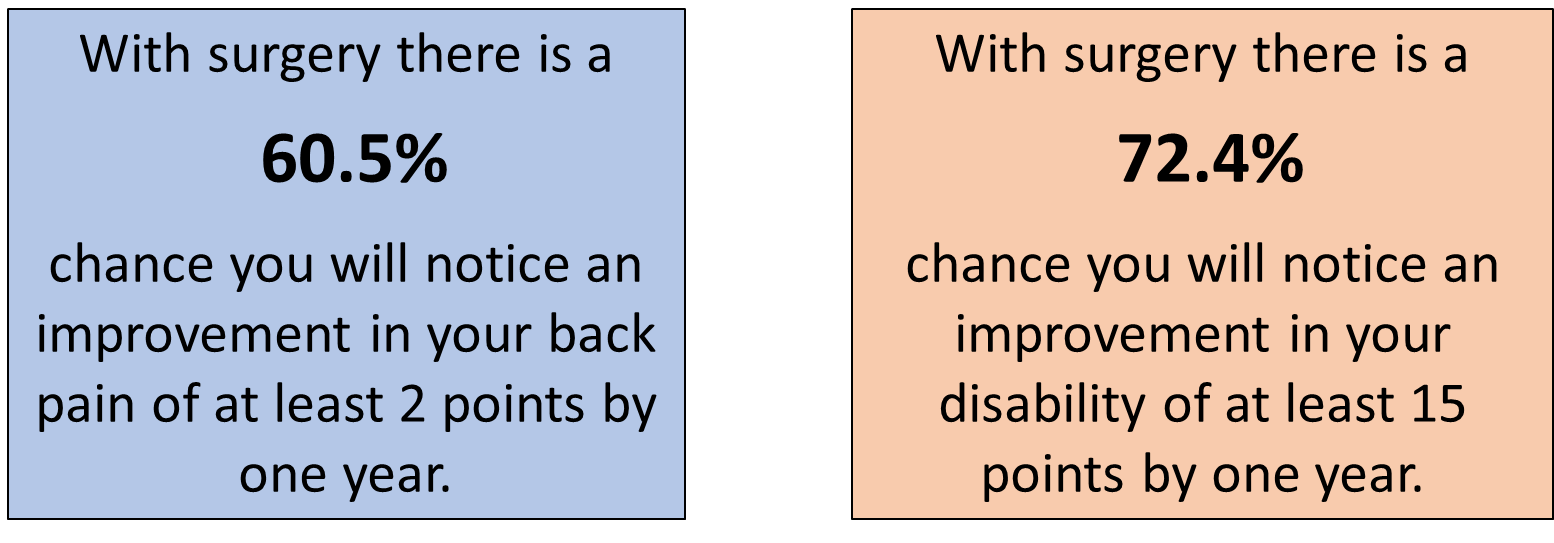

Supplement: Supplementary file 1 — Supplementary Material 1 [file 12911_2023_2234_MOESM1_ESM.docx]
