# Supplementary material for: Leveraging web-based prediction calculators to set patient expectations for elective spine surgery: a qualitative study to inform implementation
Source: BMC Med Inform Decis Mak. 2023 Aug 3;23:149. doi: 10.1186/s12911-023-02234-z (PMC10399016; doi:10.1186/s12911-023-02234-z)
Supplement: Supplementary file 2 — Supplementary Material 2 [file 12911_2023_2234_MOESM2_ESM.docx]

**Additional File 2.Semi-structured Interview Guide for Surgical Outcomes Predictive Calculator – Surgeon/Other Health care provider/Administrator [A]/Health IT Professional [IT]**

***[Show calculator here]***

**Perceived usefulness/acceptability**

1. How do you feel this calculator would improve or detract from surgical decision-making? [A]
2. How would you use the calculator with patients?

**Workflow and communication**

1. When should patients complete this calculator (complete at home and bring to their appointment, or during the clinic visit)?
   1. *What are potential benefits and drawbacks to each approach?*
2. The cervical calculator will require some data to be entered by the health care provider. Who should enter these data?
3. What disruptions to the clinical workflow would you anticipate with use of this calculator?
   1. Would you anticipate more or less disruptions depending on whether the patient completes at home, waiting room, treatment room, etc…?
4. What other modifications to the workflow may be necessary? [IT]

**Clinical content**

1. This tool will use information that is entered by the patient (e.g., symptoms, disability, etc). What concerns would you have about the validity or quality of this self-reported information? [IT]
   1. *How would these concerns influence your perception of the tool’s usefulness for decision-making?*

**Computer Interface**

1. How should patients/providers interact with the calculator (tablet, phone, computer)? [IT]
2. How and where should results be displayed to optimize its utility?
   1. *IT: What are options for displaying results? [IT only]\*
3. What other information would you want displayed along with the calculator results?
   1. *IT: What other information could be displayed along with results? [IT only]*

**Resource needs and constraints**

1. What **provider** training/education or materials (e.g., user guide, communication scripts) would help facilitate implementation and use of the calculator? [A, IT]
2. What **patient** education or materials (e.g., user guide, links to other resources, definitions) would help facilitate implementation and use of the calculator? [A, IT]
3. What resources do you think would be required to implement the calculator (e.g., money, training, education, physical space, time, etc.)? [A,IT]
4. How should we market the calculator **to patients** within the Spine Center and more broadly within VUMC/DUHS? [A]
5. What are some ways we can remind providers to use the calculator during clinic?

**Internal Organizational Policies, Procedures, and Culture**

1. What aspects of the culture at VUMC/DUHS could be a barrier/facilitator for implementation of the calculator? [A, IT]
   1. *How receptive to change do you believe administration and clinicians would be in implementing this calculator?*

**External Rules, Regulations, and Pressures**

1. What policies or regulations outside of VUMC/DUHS (e.g., governmental, insurance, etc) do you think could impact implementation of the calculator? [A, IT]
2. Do you think patients could perceive inclusion of demographic information in the calculator / in surgical decision making as discriminatory (i.e., they would have concerns the information could be used to deny preferred treatment)?
   1. *If a patient did voice concerns along these lines, how would this be ideally handled?*
