# Supplementary material for: Leveraging web-based prediction calculators to set patient expectations for elective spine surgery: a qualitative study to inform implementation
Source: BMC Med Inform Decis Mak. 2023 Aug 3;23:149. doi: 10.1186/s12911-023-02234-z (PMC10399016; doi:10.1186/s12911-023-02234-z)
Supplement: Supplementary file 3 — Supplementary Material 3 [file 12911_2023_2234_MOESM3_ESM.docx]

**Additional File 3. Semi-structured Interview Guide for Surgical Outcomes Predictive Calculator – Patient**

**Background and current activities**

1. When making decisions about whether to undergo spine surgery, what information is important for you to know before making your decision?
   1. *How would you rank the importance of this information? (i.e., which factors are most important?)*
   2. *How important is the surgeon’s opinion in comparison to what you have mentioned?*
   3. *What other factors influence your decision to undergo surgery?*

***[Show calculator here]***

**Perceived usefulness/acceptability**

1. How would you use the information provided by the web-based program (i.e., predicted probability for pain and disability after surgery)?
   1. *How comfortable would you feel interpreting a probability of improvement after surgery (e.g., a 65% chance of meeting a certain threshold for improvement by 1 year following surgery)?*
2. How could the web-based program help you discuss your surgical decision with your surgeon?
3. Is there anything else that would help you discuss the web-based program results with your surgeon?
4. Overall, what do you think are the benefits of using the web-based program and the probabilities provided?
5. What do you think are some potential drawbacks of using the web-based program?
   1. *Would you perceive inclusion of demographic information in the calculator / in surgical decision making as discriminatory (i.e., you would have concerns the information could be used to deny preferred treatment)?*
   2. *What are your thoughts on some ways this web-based program might be harmful to patients?*
   3. *Should patients be given the option to opt-out of using the web-based program?*

**Workflow and communication**

1. When during the clinical encounter would you want to see results of this web-based program? At the beginning of the conversation? After all options have been presented?
2. How do you feel surgeons should talk to you and other patients about this web-based program?
   1. *What words or phrases might help you understand the purpose of this web-based program?*
   2. *What words or phrases could be confusing or off-putting?*

**Computer Interface**

1. How could the format and output be improved so that it is more useful to you and other patients?

**Resource needs and constraints**

1. What additional information would you want to see or know alongside the results of this web-based program to help you make you decision about surgery?
